# The role of external beam radiation therapy in the management of thyroid carcinomas: A retrospective study in Iran Cancer Institute

**DOI:** 10.1002/cnr2.1652

**Published:** 2022-06-12

**Authors:** Ebrahim Esmati, Alireza Aleyasin, Reza Ghalehtaki, Fatemeh Jafari, Farshid Farhan, Mahdi Aghili, Peiman Haddad, Ali Kazemian

**Affiliations:** ^1^ Department of Radiation Oncology, Cancer Institute, Imam Khomeini Hospital Complex Tehran University of Medical Sciences Tehran Iran; ^2^ Radiation Oncology Research Center (RORC), Cancer Research Institute Tehran University of Medical Sciences Tehran Iran; ^3^ School of Medicine Tehran University of Medical Sciences Tehran Iran

**Keywords:** endocrine cancer, external beam radiotherapy, head and neck cancer, thyroid cancer

## Abstract

**Background:**

Thyroid cancers are histologically classified into three types; differentiated thyroid carcinoma (DTC), medullary thyroid carcinoma (MTC), and anaplastic thyroid carcinoma (ATC). Among the several therapeutic strategies for treatment and management of thyroid cancer, surgical resection in combination with radioactive iodine therapy (RAI) is indicated for moderate to high‐risk differentiated thyroid cancer (DTC) patients‐ according to current guidelines. However, external radiation therapy (EBRT) can be a viable alternative treatment option for these patients and scarce evidence is available regarding the efficacy and effectiveness of EBRT on thyroid cancer.

**Aim:**

This study aims at evaluating the role of EBRT in the management of thyroid carcinomas.

**Methods and Results:**

In this retrospective cohort study, the records of 59 patients with thyroid cancer were accessed who were treated by EBRT from 2008 to 2016. The indications for EBRT included unresectable primary (definitive) or loco‐regional recurrences (salvage) not suitable for RAI, palliation for local disease or metastatic foci (palliative), and the adjuvant treatment for suspected residual disease following resection. Progression‐free survival (PFS) and overall survival (OS) were calculated for different types of cancer. PFS was measured from the start of EBRT to the last uneventful follow‐up, recurrence, or death. Kaplan–Meier model was used for the survival analysis. Fifty‐nine patients were evaluated. The histopathology of the tumors was differentiated and poorly‐differentiated, medullary and anaplastic thyroid carcinomas in 22 and 6, 15 and 16 patients, respectively. Twenty‐seven patients received external beam radiotherapy (EBRT) as adjuvant therapy and 18 of the cases as palliative therapy while the remaining received salvage or definitive primary EBRT. The stage of patients' cancer was as follows: stage II in 3 and III in 1, IVA in 18 and IVB in 18 and IVC in 19. Stage‐based median overall survival was 26 months for IVA, 44 for IVB, and 29 for IVC. The median PFS was 18, 22 and 21 months for stages IVA, IVB and IVC, respectively.

**Conclusion:**

Based on our findings, EBRT may still play a role in the management of patients with thyroid carcinoma and should be considered in the armamentarium against thyroid cancers.

## 1 INTRODUCTION

Thyroid carcinomas are one of the most common endocrine cancer, which shows an increase in prevalence in recent years.^1^ Based on Iranian cancer institution, incidences of thyroid cancer is 1.8%.^2^ Thyroid cancers are histologically categorized into three major groups as differentiated thyroid carcinoma (DTC), medullary thyroid carcinoma (MTC), and anaplastic thyroid carcinoma (ATC). DTCs include papillary, follicular, and Hurthle cell carcinomas, accounting for about 80%, 11%, and 3% of all thyroid cancers, with 10‐years overall survival (OS) rates of 93%, 85%, and 76%, respectively.^3^ MTC accounts for 5% to 10% of all cases of thyroid cancers.^4^, ^5^ Five‐year OS of patients with MTC is 86%, and in 10 years, it is 65%.^6^ Anaplastic Thyroid Carcinoma (ATC) is a rare and invasive disease that accounts for less than 2% of all thyroid cancers and has a median survival of 5 months with a 20% one‐year OS.^7^, ^8^ Different patterns of behavior of DTC, MTC, ATC, and other types of thyroid cancer require different therapeutic strategies.

There are different international guidelines for the management of thyroid carcinomas.^9^ However, it seems that the standard treatment for DTCs is surgical resection followed by radioactive iodine (RAI) in moderate to high‐risk patients.^10^ According to the statement of the American Head and Neck Society, EBRT is recommended for patients who are older than 45 years and have gross residual or unresectable locoregional disease or patients with greater probability of microscopic residual disease and lower response to RAI. EBRT is not indicated as an adjuvant therapy following total reaction of the gross disease. Also, EBRT is not indicated as an adjuvant therapy upon cervical lymph node involvement alone.^11^ The use of external radiation therapy (EBRT) has been endorsed in thyroid cancer in some guidelines. Nevertheless, it is rarely performed and is limited to a small group of patients with a suspected residual disease such as those with the extrathyroidal extension (ETE) following thyroidectomy.^7^, ^12^ The evidence of EBRT usefulness is mostly obtained from retrospective single‐center studies.^13^ However, one of the rarely available randomized clinical trials examined thyroid cancer patients and showed that EBRT was generally well tolerated with promising loco‐regional control.^14^, ^15^

EBRT has also been recommended in patients with MTC or poorly differentiated thyroid cancer who are not suitable for RAI due to none or low avidity to ^131^I.^16^ Therefore, the role of EBRT in patients with DTC and MTC remains elusive.

As stated, there is limited data on the role of EBRT in thyroid cancer, and the need for further study to achieve conclusive results is obvious. More studies are required to accumulate the experiences for EBRT utilization in thyroid carcinoma. Besides, considering the national published literature on thyroid cancer patients admitted to the radiation oncology wards in Iran, we have decided to conduct an analytical descriptive study in this regard.

## 2 METHODS

### 2.1 Study design and participants

In this retrospective cohort study, the archives of the radiation oncology ward were accessed for case finding. We looked for patients with pathologically confirmed thyroid cancer treated by EBRT between 2008 and 2016 in cancer institute, which is affiliated to Tehran University of Medical Sciences. We excluded patients with thyroid pathologies other than carcinoma. Patients who did not come back or answer their phones for follow up after completion of radiation therapy were excluded as well. The design of our study was reviewed and approved by the institutional review board and ethics committee. Written informed consent is obtained routinely from all admitted patients allowing use of their medical information for research purposes.

### 2.2 Data collection

Demographic data, time of presentation of the disease symptoms, imaging findings, tumor histology, stage of the disease, type of chemotherapy (regimen, duration, and the number of courses), and type of radiotherapy (beam energies, portals, total dose and the number of fractions), time of last follow‐up, relapse or death of patients were collected and recorded in specific forms. Phone calls were made to obtain information about the patients who did not return for follow‐up sessions.

### 2.3 Histological definition and staging

We included all patients with thyroid carcinoma in our study. We categorized patients into four different groups based on histopathology, including differentiated, poorly‐differentiated, undifferentiated (ATC), and MTC. The differentiated group comprised all variants of PTC and FTC except for insular and Hurthle cell carcinomas that were included in the poorly‐differentiated group. The histopathological definition was made at the time of EBRT. Thus, some ATCs were de novo, and some were converted from the earlier PTC or FTC. We used 7th edition of American Joint Committee of Cancer for staging of our patients' cancer.

### 2.4 Treatment indications and design

We used three‐dimensional conformal radiation therapy (3DCRT) technique for majority of patients. The indications for treatment with EBRT were subdivided into four main categories: unresectable primary disease, unresectable recurrence, adjuvant treatment for residual disease, and palliative radiotherapy. The patients with unresectable primary and recurrent diseases were not candidates for surgical resection or RAI due to the bulk of the disease and invasion to surrounding organs. These patients underwent definitive radiation therapy followed by systemic targeted therapy by small‐molecule tyrosine kinase inhibitors such as sunitinib and sorafenib. The clinical target volume (CTV) included the gross disease and neck nodes located in levels II to VI and superior mediastinal nodes up to carina.^17^ The gross disease received 60 to 70 Gy at the discretion of the attending radiation oncologist based on the tolerance of organs at risk (OAR). The elective nodes received 46 Gy. With 6–18 MV energies, the photon beams were arranged as the 90% to 95% isodose volumes covered 95% of the PTV.

Adjuvant EBRT was indicated for all ATCs and those DTCs who had confirmed residual disease based on pathological and radiological reports. For the adjuvant EBRT, the target volumes included the thyroid bed, including suspicious areas of residual disease and bilateral nodes, both elective and pathologically involved. The total dose was 60 Gy or higher at the discretion of the attending radiation oncologist and based on the tolerance of OARs. Adjuvant EBRT, in PTC and FTC, was performed following RAI therapy to decrease the risk of possible stunning of the residual follicular cells. The adjuvant EBRT for ATC was delivered with concomitant chemotherapy.

Patients with the metastatic disease not amenable for curative therapy underwent palliative EBRT for the symptomatic local disease in neck or distant metastasis in the bone and brain. The total prescribed dose was in the order of 20 to 40 Gy in 5 to 15 fractions.

### 2.5 Outcomes and analysis

Progression‐free survival (PFS) was measured from the start of EBRT until the disease progression or death. Overall survival (OS) was also measured from the start of EBRT to death of the patients. The data has been gathered and analyzed via SPSS V.20.0.0. Descriptive statistics was used to describe the basic features of the data in our study. Kaplan Meyer survival analysis was and Cox hazards test were also performed. P‐values less than 0.05 were considered statistically significant.

## 3 RESULTS

### 3.1 Baseline characteristics

In our study, a total of 59 people were analyzed. Of these, 36 (61%) were male. The mean age of patients was 57.95 ± 15.91 years. The histopathology of the tumors was differentiated, poorly‐differentiated, MTC, and ATC in 22 (37.3%), 6 (10.2%), 15 (25.4%), and 16 (27.1%) patients, respectively. Diagnosis of the differentiated and poorly‐differentiated cancers was PTC and FTC, Hurthle cell, and insular carcinoma in 22 (37.3%) and 4 (6.8%), 2 (3.4%), and two patients (3.4%), respectively. Of the undifferentiated cases, two were converted from DTC, and the remainder were de novo ATCs. The stage of patients' cancer was as follows: stage II in 3 (5.1%), III in 1 (1.7%), IVA in 18 (30.5%) and IVB in 18 (30.5%) and IVC in 19 (32.2%).

The clinical and tumor characteristics based on the indication for EBRT are summarized in Table 1.

**TABLE 1:** Clinical and treatment characteristics

		Definitive	Adjuvant	Salvage	Palliative
Age		65 (±23.4)^a^	54 (±16.6)	62 (±10.0)	58 (±13.5)
Gender	Male	5 (71.4)^b^	15 (55.6)	4 (57.1)	12 (66.7)
	Female	2 (28.6)	12 (44.4)	3 (42.9)	6 (33.3)
Histopathology	DTC	4 (57.1)	3 (11.1)	4 (57.1)	11 (61.1)
	PDTC	1 (14.3)	1 (3.7)	1 (14.3)	3 (16.7)
	MTC	0	10 (37)	2 (28.6)	2 (28.6)
	ATC	2 (28.6)	13 (48.1)	0	1 (5.6)
Staging	II	0	0	0	3 (16.7)
	III	0	1 (3.7)	0	0
	IVa	0	17 (63)	1 (14.3)	0
	IVb	6 (85.7)	8 (29.6)	3 (42.9)	1 (5.6)
	IVc	1 (14.3)	1 (3.7)	3 (42.9)	14 (77.8)
History of RAI		0	3 (11.1)	4 (57.1)	5 (27.8)
Median RT total dose		60 (40–60)^c^	60 (54–60)	60 (56–66)	30 (30–40)

^a^ Mean (standard deviation).

^b^ Number (%).

^c^ Median (IQR).

Proportion of patients undergoing adjuvant, palliative, salvage or definitive EBRT were 27 (45.8%), 18 (30.5%), 7 (11.9%), and 7 (11.9%), respectively. Four palliative cases received RT for local disease while the other 14 cases received RT for the metastatic foci in bone or brain. The histological distribution of tumors within each category of EBRT indication is shown in Table 2. The majority of tumors were differentiated in definitive, salvage, and palliative categories. However, in the adjuvant category, the majority were undifferentiated tumors.

**TABLE 2:** The EBRT indication in patients with each histopathological subtype

	Diagnosis at EBRT				
EBRT indication	Differentiated	Poorly differentiated	Anaplastic de novo	Anaplastic conversion	MTC
Definitive	4 (18.2)^a^	1 (16.7)	2 (14.3)	0 (0)	0 (0)
Adjuvant	3 (13.6)	1 (16.7)	11 (78.6)	2 (100)	10 (66.7)
Salvage	4 (18.2)	1 (16.7)	0 (0)	0 (0)	2 (13.3)
Palliative	11 (50)	3 (50)	1 (7.1)	0 (0)	3 (20)
Total	22 (100)	6 (100)	14 (100)	2 (100)	15 (100)

^a^ Number (%).

### 3.2 Outcomes

In the follow‐up period, 22 patients died, while 33 patients suffered from the disease's progression. The median PFS for the patients who underwent definitive, adjuvant, salvage, and palliative EBRT was 25, 22, 16, and 12 months, respectively. The hazard ratios, based on multivariate Cox regression analysis, for OS and PFS are depicted in Table 3. None of differences were statistically significant based on the 95% CI provided in the tables.

**TABLE 3:** The hazards for histological type, stage and RT indication for OS and PFS

		Median OS (months)	Adjusted HR (CI95%) for OS	Median PFS (months)	Adjusted HR (CI95%) for PFS
RT category	Definitive (Unresectable Primary)	26	Ref.	25	Ref.
	Adjuvant Primary	Not reached	0.7 (0.08–6.25)	22	1.5 (0.25–9.45)
	Salvage (Unresectable Primary)	26	1.6 (0.11–23.30)	16	5.5 (0.72–41.48)
	Palliative	28	5.6 (0.30–102.88)	15	6.9 (0.67–73.84)
Stage	IVA	26	Ref.	18.6	Ref.
	IVB	44	0.4 (0.09–2.15)	22	1.1 (0.38–3.68)
	IVC	29	0.1 (0.01–2.00)	21	0.4 (0.06–2.69)
Histological type	Differentiated	29	Ref.	23	Ref.
	Poorly‐differentiated or Undifferentiated	Not reached	0.8 (0.16–3.53)	12	1.4 (0.47–4.22)
	MTC	40.5	0.5 (0.09–3.32)	26	0.6 (0.17–2.43)

Stage‐based median overall survival rates were as follows: 26 (CI 95%: 0–64) for IVA, 44 (CI 95%: 6–83) for IVB and 29 (CI 95%: 12–46) for IVC. The median PFS rates were 18 (CI 95%: 0–39), 22 (CI 95%: 14–30) and 21 (CI95%: 12–30) for stages IVA, IVB and IVC, respectively.

**FIGURE 1:**
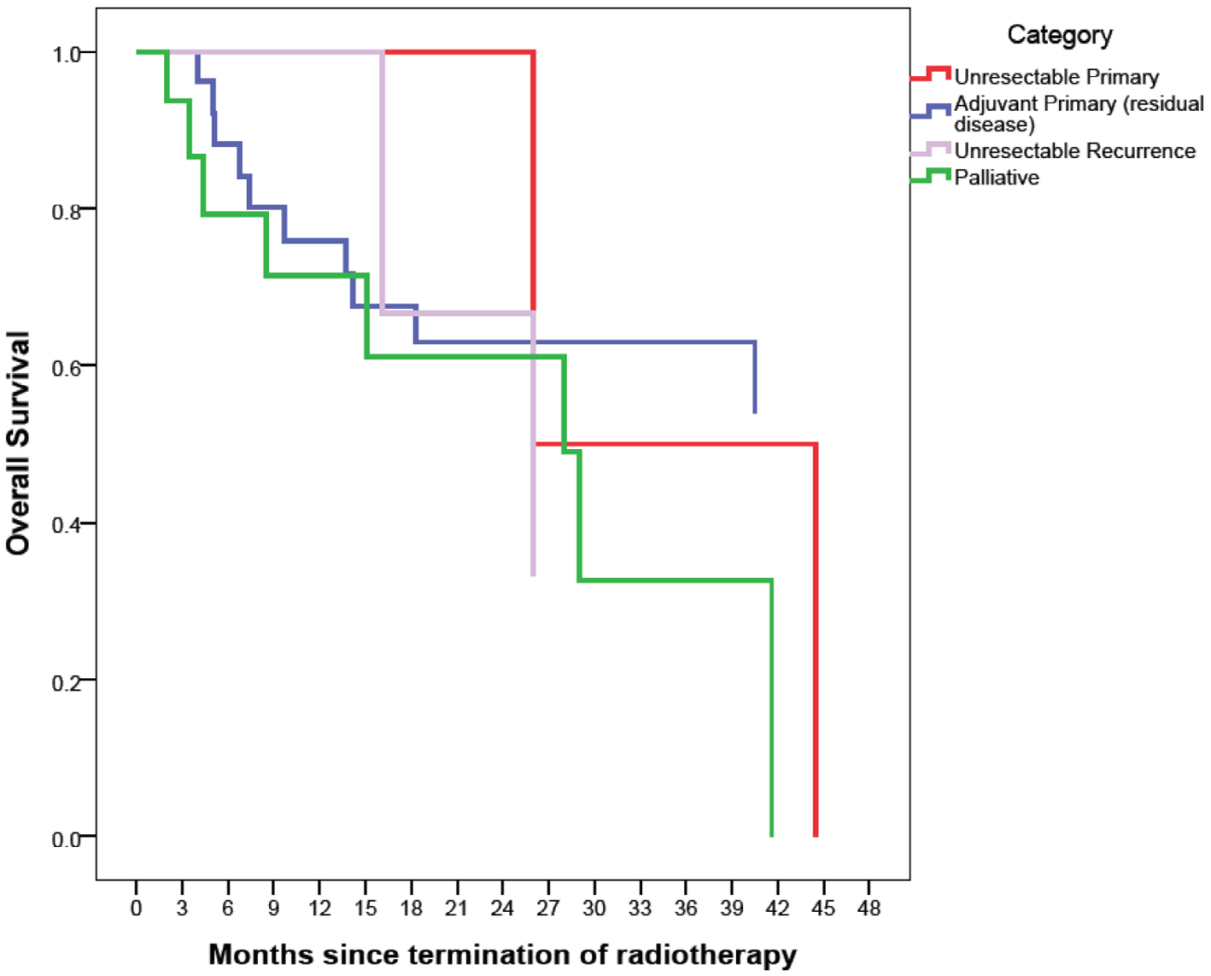
Overall survival based on RT category with Kaplan–Meier method. The reference line points out to the median survival

**FIGURE 2:**
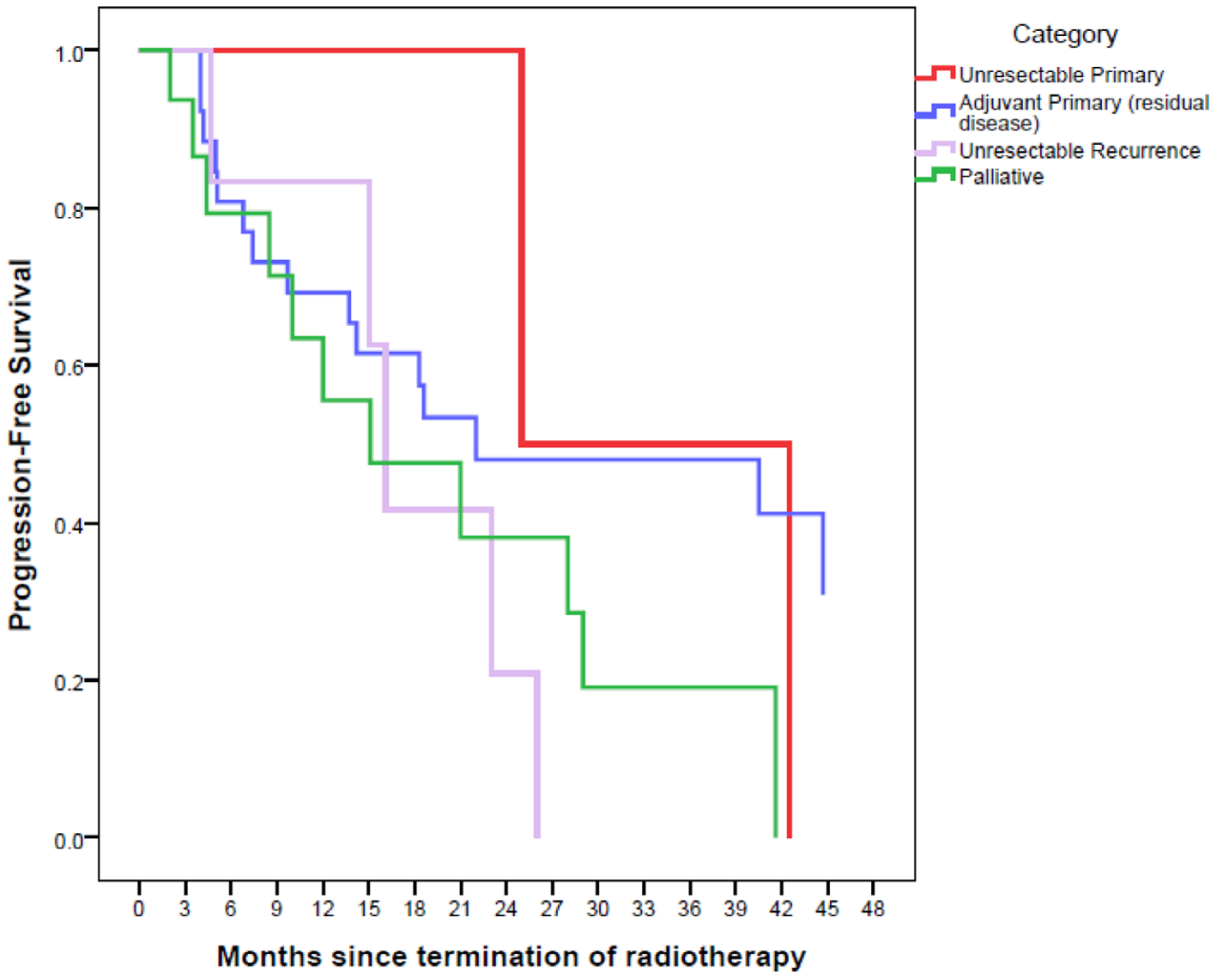
Progression‐free survival based on RT category with Kaplan–Meier method. The reference line points out to the median survival

**FIGURE 3:**
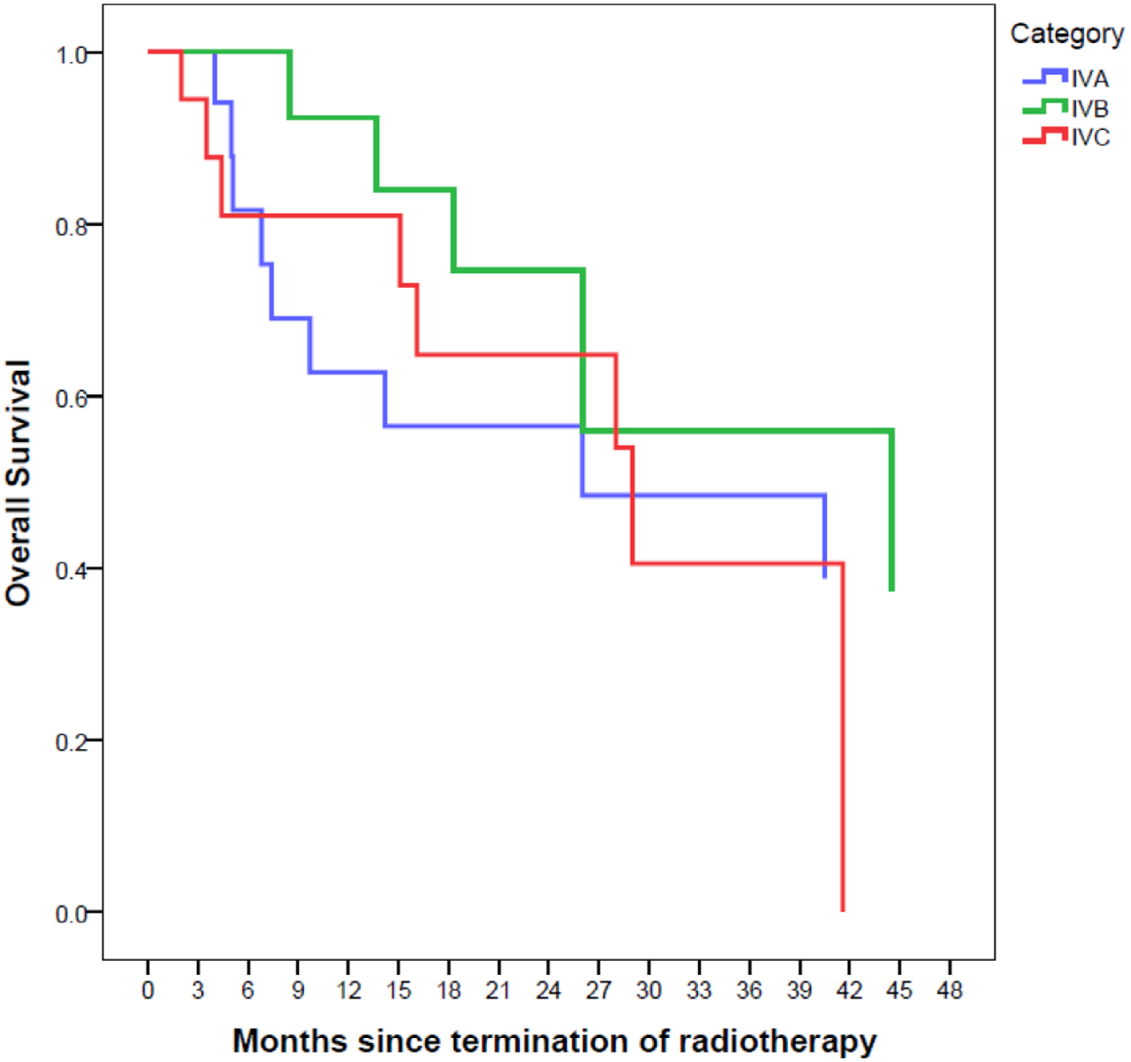
Overall survival based on stage with Kaplan–Meier method. The reference line points out to the median survival

**FIGURE 4:**
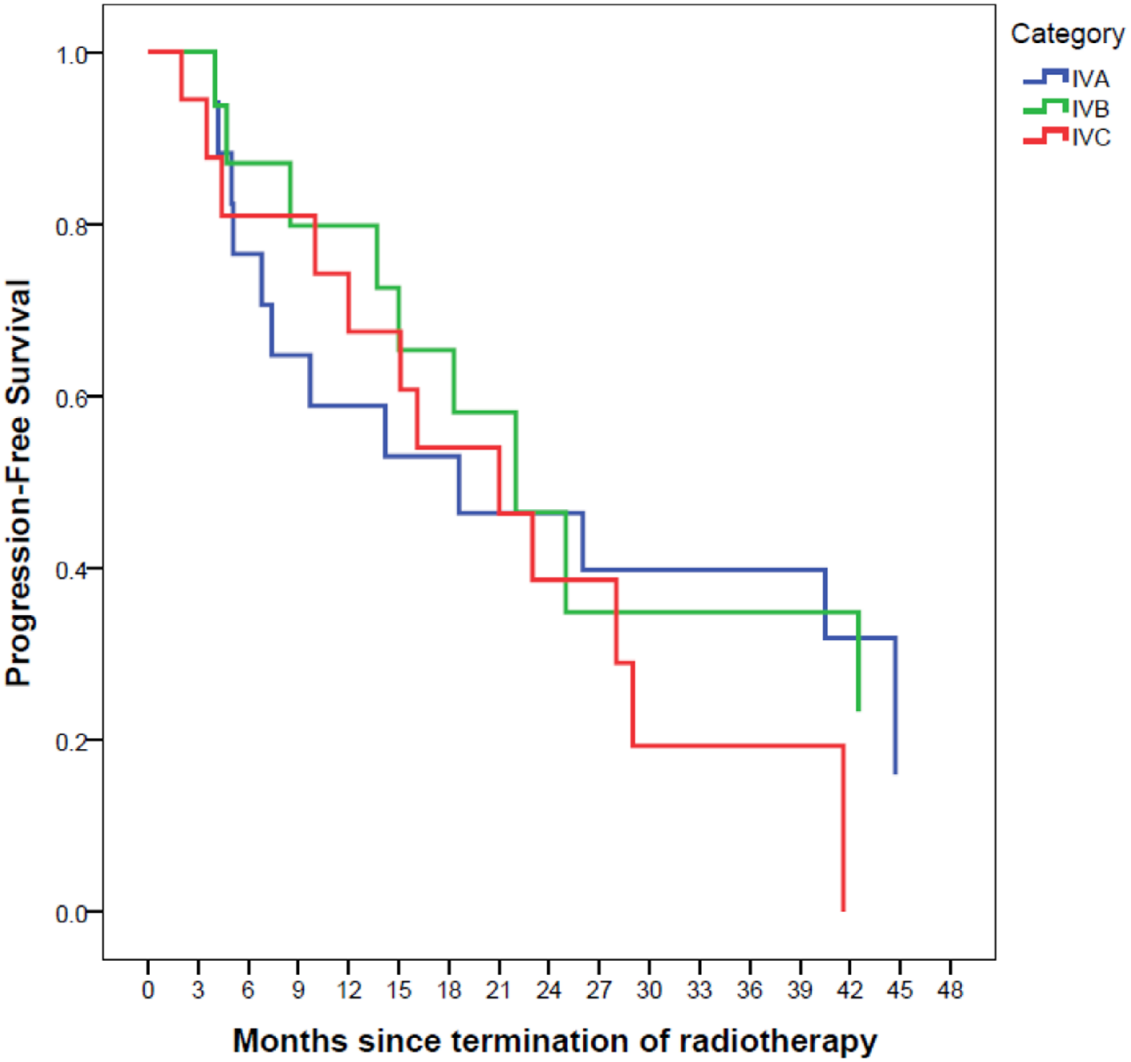
Progression‐free survival based on stage with Kaplan–Meier method. The reference line points out to the median survival

**FIGURE 5:**
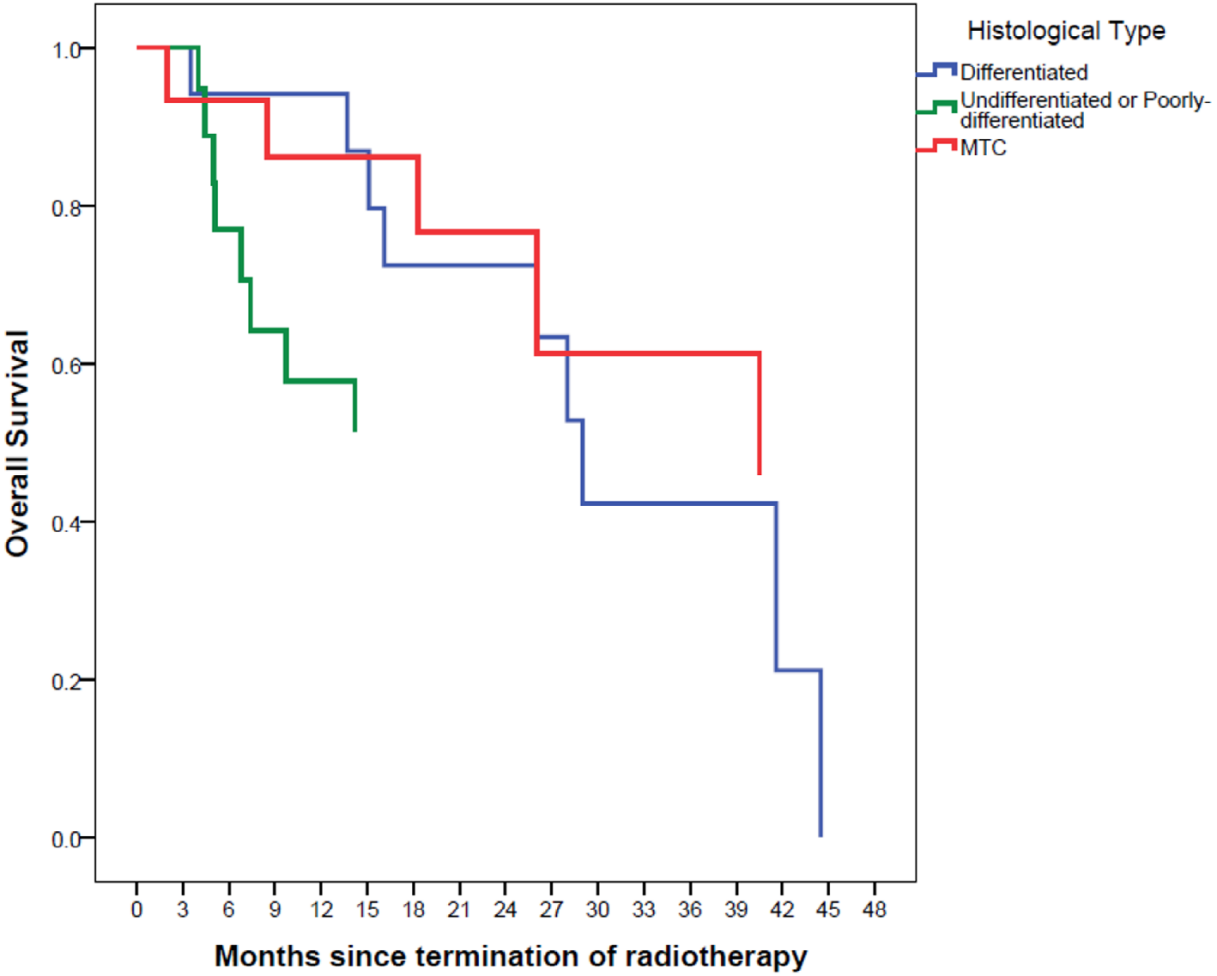
Overall survival based on histological type with Kaplan–Meier method. The reference line points out to the median survival

**FIGURE 6:**
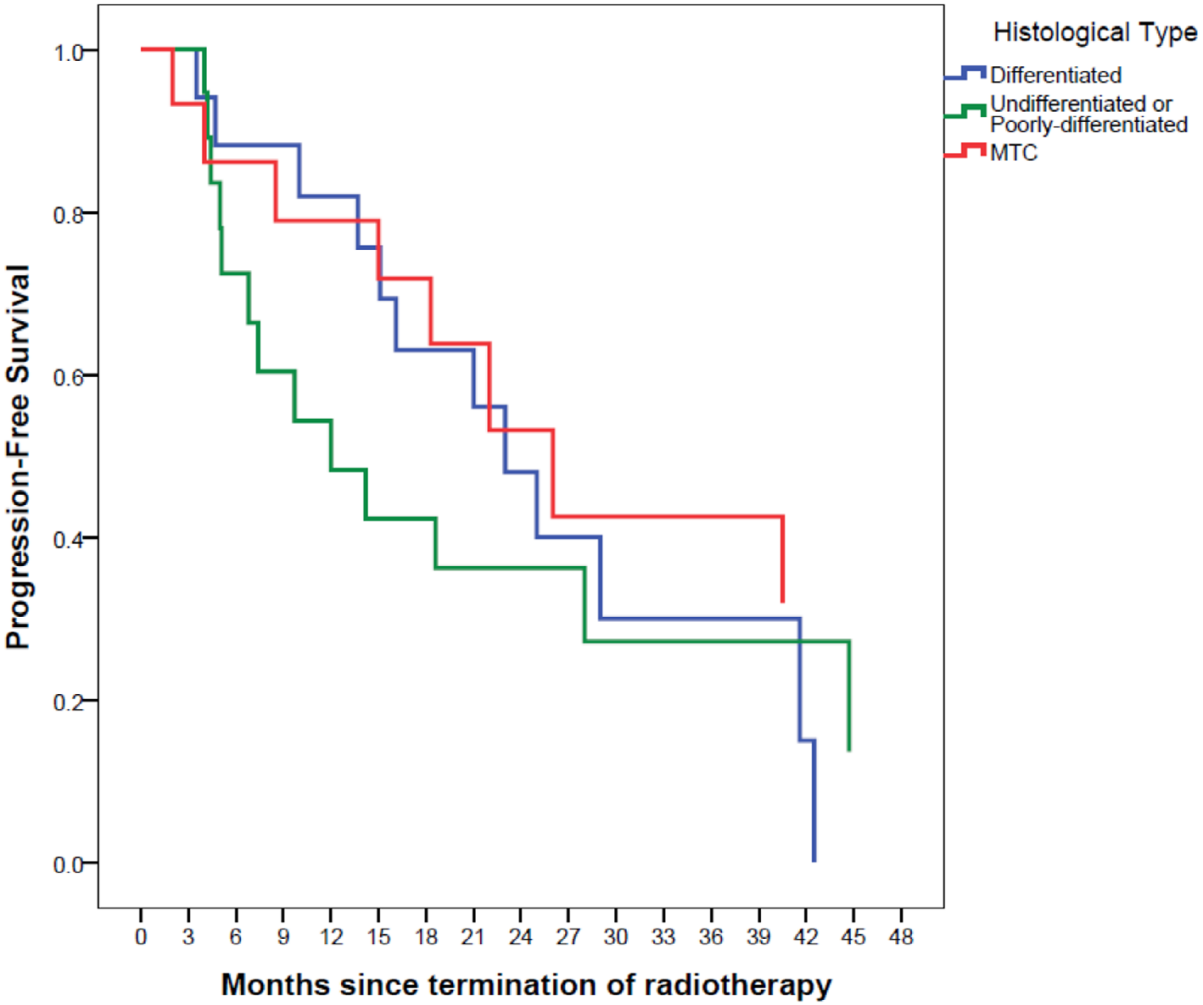
Progression‐free survival based on histological type with Kaplan–Meier method. The reference line points out to the median survival

The Kaplan–Meier survival curves are depicted in Figures 1, 2, 3, 4, 5, 6.

## 4 DISCUSSION

Overall, there is no consensus among the studies regarding the efficacy and applicability of EBRT on different stages, different settings, and histological subtypes of thyroid cancer. This represents the lack of clinical evidence supporting the efficacy of EBRT and the great need for further long‐term prospective multicenter studies.^18^

The distribution of histological subtypes in our patient cohort was different from general population findings.^19^ As a whole, DTC that usually absorb RAI shares the biggest contribution^20^, ^21^ while in our cohort only near a third of patients had ^131^I absorbing DTC. The use of EBRT for treatment of differentiated thyroid carcinomas has declined overtime since overwhelming evidence for usefulness of RAI therapy and more comprehensive surgeries. Nevertheless, sometimes EBRT is the only available option to help patients with DTC for better quality of life and relieve of pain or airway obstruction with the primary or metastatic disease. A minority of patients with unresectable primary or recurrent disease in the neck would also benefit from addition of EBRT to RAI and surgery. Aside from DTC, the main indication for EBRT in thyroid cancers is for eliminating residual disease in the neck following resection of ATC or MTC that have no other effective therapy. Anaplastic thyroid carcinoma does not take up ^131^I, hence, EBRT and maximal safe surgical resection are the treatment modality of choice. For the MTC, also there is not any available therapy other than surgical resection and due to the high probability of residual disease in the bed of surgery and neck lymph nodes, EBRT seems to be effective. These reasons explain why the distribution of histological subtypes are different in our cohort than general population.

The setting of treatment was not a predictor of survival in our cohort. Albeit, patients who received EBRT with palliative intent had the highest HR for death that was predictable. This finding is due to the selection bias. Those patients who underwent adjuvant EBRT mainly had ATC or MTC while those undergoing salvage EBRT were those with more aggressive poorly‐differentiated thyroid cancer or multiple recurring PTCs with overloaded ^131^I or unresectable bulky PTC. This is the main problem in thyroid cancer patients undergoing EBRT that are a heterogenous population. EBRT can be used as adjuvant treatment after surgical resection or as an initial treatment in unresectable cases.^22^

The survival of patients with various stages from IVa to IVc was not significantly different. This finding seems to be contrary to what is expected. The stage is the main prognostic factor at the diagnosis and before commencing any treatment. However, many patients in our cohort were heavily pretreated with multiple surgeries and repeated RAI therapy. This history would fade much of the prognostic significance of the stage. So, the staging at the time of external beam radiotherapy would not add additional prognostic information other than more robust factors such as EBRT indication or histological type. In addition, the majority of patients in our cohort had stage IV disease and we know that the difference between subcategories of a given stage is not so much evident.

Usually, patients who are recommended to receive EBRT, suffer from severe and aggressive diseases that do not respond to RAI and cannot be resected.^11^ Although due to the heterogeneity of patients in our cohort that makes it difficult to reach a firm conclusion, we could point to some facts that suggest the benefit of EBRT. The median survival of our patients was something between 22 to 44 months which is a relatively long survival with this aggressive form of the disease. Albeit some of this long survival is due to the inherent characteristics of thyroid cancer and other treatment modalities, but this could also be in part due to the beneficial effect of EBRT especially in the poorly‐diff and anaplastic and medullary thyroid cancers. Locking at the Kaplan–Meier survival curves we notice that after the initial rapid decline in the survival fraction, a proportion of patients with MTC and ATC experience a long survival without disease recurrence (Figures 5 and 6). This effect of EBRT has been emphasized on elsewhere. In a previous study, 68 Korean patients with EBRT experienced a decline in recurrence rate from 51% to 8% in papillary thyroid cancer.^23^ One study on patients with advanced papillary thyroid cancer showed that EBRT alone, RAI alone, and EBRT plus RAI are associated with 60%, 72%, and 88% 10‐year local failure‐free survival rates. Patients undergoing surgeries with positive margin also benefit from EBRT as the local failure‐free survival rates for EBRT alone, RAI alone, and EBRT plus RAI increased to 57%, 80%, and 90%, respectively.^24^ Another similar study on adjuvant EBRT in advanced DTC reported five‐year disease‐free survival rate of 57% for patients who were treated with EBRT and RAI. This study also indicates that there was no significant difference between those who have undergone EBRT alone and those who have undergone EBRT and RAI.^25^ However, some mainly retrospective studies showed that EBRT did not improve OS and disease‐free survival.^26^ The probable main reason for lack of benefit could be due to the selection bias that the irradiated group included a higher number of advanced‐stage patients than the non‐irradiated group.

Looking at the median survival for the patients in the palliative setting, this rate is so high compared with many cancers that justifies any symptom reducing efforts in this population. EBRT is an effective tool to relieve bone pain or local compressive symptoms.

In summary, the application of EBRT in thyroid cancers could be effective in local control on iodine‐resistant cases and palliation of metastatic conditions. In papillary thyroid cases, survival rates are high, so local control with EBRT is highly important. In anaplastic cases, since prognosis is poor and surgery is not always feasible, EBRT has an important role, and in‐operable medullary thyroid cases also benefit from EBRT in controlling the extent of the disease.

## 5 CONCLUSION

This study suggests that application of EBRT in thyroid cancers should be considered in the management of iodine‐resistant cases, palliation of metastatic thyroid conditions, and symptomatic control of thyroid cancers.

## AUTHOR CONTRIBUTIONS

**Ebrahim Esmati:** Conceptualization (equal); data curation (equal); methodology (equal); supervision (equal); writing – review and editing (equal). **Alireza Aleyasin:** Data curation (equal); formal analysis (equal); writing – review and editing (equal). **Reza Ghalehtaki:** Conceptualization (equal); methodology (equal); supervision (lead); writing – review and editing (equal). **Fatemeh Jafari:** Data curation (equal); writing – original draft (equal); writing – review and editing (equal). **Farshid Farhan:** Data curation (equal); investigation (equal). **Mahdi Aghili:** Methodology (equal); project administration (equal); supervision (equal). **Peiman Haddad:** Conceptualization (equal); resources (equal). **Ali Kazemian:** Investigation (equal); supervision (equal).

## CONFLICT OF INTEREST

The authors have stated explicitly that there are no conflicts of interest in connection with this article.

## ETHICS STATEMENT

The Research Ethics Committee at TUMS granted its ethics approval to this study. This study is in accordance with the Declaration of Helsinki by the World Medical Association.

## Data Availability

The data that support the findings of this study are available on request from the corresponding author.
